# REDUCING ANTIPSYCHOTIC USE IN LONG-TERM CARE: CONSIDERING THE ROLE OF CULTURE OF CARE AND INFORMAL COMMUNICATION

**DOI:** 10.1093/geroni/igz038.1623

**Published:** 2019-11-08

**Authors:** Andre Smith, Sue Kurucz, Tara Erb, Ruth Kampen

**Affiliations:** 1 Department of Sociology, University of Victoria, Victoria, British Columbia, Canada; 2 Island Health, Victoria, British Columbia, Canada

## Abstract

Persons living with dementia-related disorders (PwD) can experience challenging behavioural and psychological symptoms (BPSD) as their illness progresses. There is a continued reliance on antipsychotic drugs (APD) in long-term care to manage this issue despite the well-documented risks of adverse events and increased morbidity and mortality. This study examines the role of culture of care in relation to efforts at reducing inappropriate APD use in managing BPSD within long-term care. Culture of care consists of shared norms, beliefs, and cognitive frames which guide clinical practice and inform the development and implementation of care strategies. Findings were obtained from three Canadian long-term care facilities working on reducing inappropriate use of APD. Data came from interviews with 6 nurses, 18 licenced practical nurses, 14 health care assistants, 4 activity leaders, 4 directors of care, 1 chaplain, and 10 physicians. We found that direct care providers initially varied in their perceived ability to develop and use alternate care strategies with health care assistants being most concerned about safety and exposure to violence. Change involved detective work and innovative thinking in assessing possible causes of BPSD beyond psychosis, including pain and feelings of confusion. Informal reciprocal patterns of communication emerged among health care assistants to identify effective non-pharmaceutical strategies to manage BPSD. Overall, the study shows how shared beliefs in the need for and value of alternate care practices among direct care providers along with the existence of effective informal communication can contribute to successful reduction in APD use when managing BPSD in PwD.

